# Editorial: Cardiovascular sequelae of chemotherapy and radiotherapy in cancer survivors: current evidence and perspectives

**DOI:** 10.3389/fcvm.2023.1230862

**Published:** 2023-06-20

**Authors:** Tamara Felici, Roderick Skinner, Péter Ferdinandy, Zoltan V. Varga, Antonella Lombardo, Massimiliano Camilli

**Affiliations:** ^1^ Department of Cardiovascular and Pulmonary Sciences, Catholic University of the Sacred Heart, Rome, Italy; ^2^ Department of Cardiovascular Medicine, Fondazione Policlinico Universitario A. Gemelli IRCCS, Rome, Italy; ^3^ Department of Paediatric and Adolescent Haematology and Oncology, Great North Children’s Hospital, Newcastle upon Tyne, United Kingdom; ^4^ Translational and Clinical Research Institute, Newcastle University Centre for Cancer, Newcastle University, Newcastle upon Tyne, United Kingdom; ^5^ Department of Pharmacology and Pharmacotherapy, Semmelweis University, Budapest, Hungary; ^6^ Pharmahungary Group, Szeged, Hungary; ^7^ MTA-SE System Pharmacology Research Group, Department of Pharmacology and Pharmacotherapy, Semmelweis University, Budapest, Hungary; ^8^ HCEMM-SU Cardiometabolic Immunology Research Group, Budapest, Hungary; ^9^ MTA-SE Momentum Cardio-Oncology and Cardioimmunology Research Group, Budapest, Hungary

**Keywords:** cancer survivors, cardiotoxicity, radiotherapy, cardio-oncology, cardiac imaging, early diagnosis

**Editorial on the Research Topic** Cardiovascular sequelae of chemotherapy and radiotherapy in cancer survivors: current evidence and perspectives

Cardiovascular diseases and cancer are the two leading causes of morbidity and premature death worldwide (1, 2). In the past decade, the survival of cancer patients has impressively increased, mainly through screening modalities improvement and development of novel anticancer drugs (1, 2). This inevitably brought to a growing population of cancer survivors, burdened by long-term sequelae due to chemotherapy and radiotherapy (1–3). Indeed, chest malignancies, including lung, esophageal and breast cancers, as well as lymphomas, usually require radiotherapy as part of their treatment regimen. Even though advanced radiation techniques have provided better protective effects for the heart, the risk of delayed heart disease still represents a considerable concern.

Therefore, we should take into account the potential synergic effect of anticancer agents and radiotherapy (4–6). Moon-Sing Lee et al. in their population-based study, identified 158,798 breast cancer patients and using a propensity score match of 1:1, included 21,123 patients in each left and right breast radiation cohort. Previous heart disease and anticancer agents, including epirubicin, doxorubicin and trastuzumab, were taken into account for the analysis. Patients who received left radiation demonstrated an increased risk of ischemic heart disease compared with patients who received right breast radiation. Furthermore, in those undergoing left breast radiation dose >6,040 Gray, subsequent epirubicin might show a tendency to increase the risk of heart failure, whereas doxorubicin and trastuzumab did not. Further studies appear to be needed to confirm these hypothesis-generating results.

In the arena of emerging mechanisms involved in cardiotoxicity, the inflammatory cascade seems to be a common denominator in the pathogenesis of cardiovascular events during treatment of various types of tumors. In particular, Torrente et al. conducted a single-institution retrospective study in order to assess the incidence and identify risk factors for cardiac events in patients treated with Immune checkpoint inhibitors (ICIs) for hematological and solid tumors. These agents produce a wide spectrum of inflammatory and immune-related adverse events (7), mainly due to aberrant autoreactive T-cell activation, eventually affecting any organ or tissue. Among the various forms of ICI toxicities, myocarditis brings high risk of morbidity and mortality. In this study, 378 patients were analyzed and it was found that the incidence of cardiac events (CEs) was 16.7%, during a median follow-up of 50.5 months. The multivariable analysis showed that age, history of arrhythmia or ischemic heart disease, as well as prior immune-related adverse events, were predictors of CEs. Moreover, it appeared that CEs during ICI treatment, in particular atrial fibrillation, were more common than currently appreciated. Thus, a thorough cardiovascular evaluation is strongly recommended before ICI treatment and, in high-risk patients, a closer follow-up and prompt referral to cardio-oncology specialists, as soon as cardiotoxicity is suspected. Risk stratification using a scoring system may improve the cardiovascular outcomes of patients treated with ICIs, by identifying those at higher risk for cardiovascular complications.

In the field of innovative immunological treatment, another topic of current interest is represented by chimeric antigen receptor-T (CAR-T) cells infusion for patients affected by advanced and refractory onco-hematological malignancies. CAR-T cell-related cardiovascular toxicities are now emerging as significantly affecting patients' prognosis. Mechanisms involved are still under investigation, although the aberrant inflammatory activation observed in cytokine release syndrome (CRS) seems to play a pivotal role. The most frequently reported cardiac events, observed both in adults and in the pediatric population, are represented by hypotension, arrhythmias and left ventricular systolic dysfunction, sometimes associated with overt heart failure. Therefore, there is an increasing need to understand the pathophysiological basis of these events and risk factors related to their development, in order to identify most vulnerable patients requiring a close cardiological monitoring and long-term follow-up. A multidisciplinary approach focused on the management of this selected population is now needed to optimize outcomes Camilli et al.

The inflammatory hypothesis may be also applied to the therapeutic field. Aspirin, through its anti-inflammatory effect, can inhibit tumor cell growth and prevent the development of cardiovascular diseases (8). By suppressing cyclooxygenase 1/2, some preclinical studies have found possible antitumor mechanisms in subjects affected by prostate cancer. Wei-Ting Chang et al. starting from a reported increased risk of incident coronary heart disease and myocardial infarction in association with Gonadotropin Releasing Hormone (GnRH) antagonists treatment, aimed at studying whether regular aspirin use was associated with a risk reduction of major adverse cardiovascular and cerebrovascular events (MACCEs) by using a nationwide database. In their study, they observed that, despite a higher risk of MACCEs among irregular aspirin users, after adjusting for age, cancer stage and comorbidities, there was a trend to MACCEs reduction among those who received aspirin regularly.

Among the numerous pathological changes that occur to the myocardium during cardiotoxic therapy, oedema is considered an early manifestation of myocardial damage and a precursor of cardiac dysfunction and fibrosis (9, 10). Cardiac magnetic resonance (CMR) T2 mapping technique can characterize myocardial oedema *in vivo* and potentially provide additional insights beyond functional evaluation (9, 10). In the field of gynecological malignancies, Meng-Xi Young et al. enrolled 73 cancer patients and 41 healthy volunteers. All participants underwent CMR imaging. CMR sequences included cardiac cine, T2 mapping, and late gadolinium enhancement. In patients with gynecological malignancies, myocardial oedema developed with increasing chemotherapy cycles and was associated with decreased left ventricular mass. This report represents one of the few examples of T2 mapping application in cardio-oncology and above all in the gynecological population.

At the same time, in order to confirm the ability of native T1 and T2 values in detecting and monitoring early myocardial injury of chest radiotherapy, Yaotian Tian et al. in their prospective observational study, enrolled fifteen participants who received non-anthracycline chemotherapy and chest radiotherapy, and 30 age/gender-matched controls. Cardiac magnetic resonance scans were performed within 2 days, 3 months, and 6 months after chest radiotherapy. They demonstrated that native T1 and T2 values increased at 3 months after radiotherapy, whereas LVEF showed no significant changes during the 6-month follow-up.

Rapid development of microarray and sequencing technologies has revolutionized the complexity of collecting and examining molecular data in current biomedical research. Hongyan Qian et al. aimed at understanding the differences in the molecular mechanisms involved in doxorubicin-induced acute and chronic cardiotoxicity, through mouse models and Ribonucleic acid (RNA)-sequencing data. Mice were injected intraperitoneally with doxorubicin [(20 mg/kg, once) or (5 mg/kg/week, three times)] to simulate acute and chronic cardiotoxicity. Left ventricular myocardium samples were analyzed by RNA-sequencing to identify differentially-expressed genes. *Alas1*, *Atp5g1*, and *Ptgds* revealed to be ideal biomarkers in acute cardiotoxicity, while *Hsph1* and *Vegfa* were identified as potential biomarkers of chronic toxicity. This report first provided bioinformatics and clinical evidence for differences in mechanisms of doxorubicin-induced acute and chronic cardiotoxicity.

The increasing need to understand the pathophysiological basis of cardiotoxicity and its early detection serves to identify most vulnerable patients requiring a close cardiological monitoring and, above all, a long-term follow-up (2). With improved cancer survival, non-cancer events, especially heart diseases, have become a leading cause of death in neoplastic patients and require long-term follow-up. Bei Chen et al. in the first large population-based study on the risk of fatal cardiac events in sarcoma patients, suggested that the risk of death is higher than that observed in the general population, and progressively increased with longer follow-up times. However, in patients with bone and soft tissue sarcoma, the risk of cardiac death varied mainly in patients with different histological subtypes of sarcoma and disease stages. Subgroup analyses indicated that chemotherapy increased the risk of events in patients with localized osteosarcoma, but not in those with other histological sarcoma subtypes and clinical characteristics. Thus, to mitigate the risk of death in sarcoma patients, enhanced multidisciplinary cooperation is warranted.

Similarly, Xuezhen Wang et al. explored the impact of chemotherapy on the risk of cardiac-related death in astrocytoma patients, by retrospectively evaluating astrocytoma patients diagnosed between 1975 and 2016 in the Surveillance, Epidemiology, and End Results (SEER) database. They found that age at diagnosis plays a crucial role in determining the risk of subsequent cardiac death rather than chemotherapy, highlighting that cardio–oncologists must provide comprehensive care and long-term monitoring for cancer patients, especially those at an increased cardiovascular risk.

This special issue again sheds light on the emerging role of cardio-oncology teams, always more necessary to reduce the burden of cardiovascular diseases in cancer patients through surveillance strategies implementation and cardiotoxicity management protocols.
